# The Quest for the Associative Device for Hebbian‐Like LTP—The Accidental Participant

**DOI:** 10.1002/hipo.70116

**Published:** 2026-08-05

**Authors:** Bengt Gustafsson

**Affiliations:** ^1^ Dept. Physiology, Sahlgrenska Academy University of Gothenburg Gothenburg Sweden

**Keywords:** Hebbian, hippocampus, inhibition, LTP, NMDA receptor

## Abstract

In 1985 Holger Wigström (now deceased) and I published a proposed scenario for the induction of hippocampal Hebbian‐like LTP to explain its input specificity and cooperativity. In this scenario LTP is induced by calcium influx through voltage‐dependent NMDA receptor channels co‐localized with the glutamate receptor channels (non‐NMDA) mediating the expression of LTP. This co‐localization, we argued, explained the input specificity, and the voltage dependence of these NMDA receptor channels explained the cooperativity. It will be described how this scenario emerged from our finding of a greatly facilitated LTP induction following blockade of postsynaptic inhibition, from our observation of a dendritic, possibly regenerative, event correlated with successful LTP induction, and to our recognition that these events were the result of NMDA receptor activation. Our observation of a likely synaptic co‐localization of these receptor channels with the non‐NMDA receptor channels, with the former contributing to an EPSP component with a slower onset and much longer duration, will be described. Finally, I will describe how our scenario became validated by our experiments pairing such single EPSPs with depolarization induced by injected current pulses, temporally associated with the NMDA component. This essay will conclude with some take‐home messages regarding the Hebbian‐like LTP as learned from these experiments: this form of synaptic learning most likely only occurs during disinhibition, its longevity strongly depends on the induction strength from a few minutes and upwards, with a strong induction event it can operate as a one‐shot potentiation, and its time window for association may remain several 100 ms. Lastly, Hebbian‐like LTP likely evolved to associate input events rather than an input event and somatic spike activity.

## 1 Introduction

In early 1985, Holger Wigström (who sadly passed away in 2024) and I published a paper in Acta Physiologica Scandinavica on LTP induction (Wigström and Gustafsson 1985b). Based on our experimental data on hippocampal synapses we proposed that LTP is basically not a post‐tetanic form of synaptic plasticity but can be induced by a single presynaptic transmitter release event. This single release would (at every spine) result in glutamate binding to two types of postsynaptic receptors, a non‐NMDA (AMPA) type mediating the normally observed EPSP, and an NMDA type producing a normally silent EPSP with slower onset and much slower decay. Strong postsynaptic activity applied during this tentative NMDA EPSP should open the voltage‐dependent and calcium‐permeable NMDA receptor channels and result in postsynaptic calcium influx and LTP induction. The restriction of calcium influx into only activated spines together with the voltage dependence of the NMDA receptor channels would explain the input specificity of LTP and the cooperativity requirement for LTP, respectively.

Later in the spring that same year (1985) we submitted a paper in the form of a Letter to Nature showing that LTP is induced under conditions when a single test EPSP (in the hippocampal CA3‐CA1 connection), evoked at low (0.2 Hz) frequency, is paired with postsynaptic activity elicited by a pulse of injected current through the intracellular microelectrode, and that this LTP was blocked in the presence of an NMDA receptor antagonist. These results strongly suggested that our proposal disclosed the elusive associative device underlying the Hebbian‐like character of LTP induction. They also definitely distinguish LTP in these synapses from a post‐tetanic facilitation/potentiation process. However, after some back‐and‐forth communication with Nature for almost half a year this article was ultimately rejected. In 1989 Holger and I received the Alden‐Spencer Award (Columbia University) for this work, an award established in 1978 to recognize scientists who have made outstanding research contributions to the field.

## 2 Personal Background

So, who were we? Holger Wigström went to school in Jönköping, a major city in the central part of southern Sweden (known as the Bible belt of Sweden). He then moved to Göteborg, (Sweden's 2nd largest city) on the west coast to become a civil engineer specialized in physics and mathematics at Chalmer University of Technology. Thereafter, in the early‐mid 1970s he became involved in a Ph.D. project dealing with neural network models based on a Hebbian learning rule (Wigström 1973, 1974, 1975). Following this theoretical work, he went to Per Andersen's lab in Oslo to learn how to use the in vitro hippocampal slice preparation to study LTP (Andersen et al. 1980, 1977). He returned to Göteborg in the late 1970s to set up an in vitro hippocampal slice preparation at our neurophysiology section at the Medical Faculty in Göteborg. Later (1989), in a birthday letter to Per Andersen, Holger indicated that he had been rather doubtful regarding the value of studying brain processes in such a diminutive structure as a slice (~400 μm thick) but was happy that Per had convinced him of its value, having now recognized the slice as being a “universe of its own and we are travelers whose journey has just begun.”

With two older brothers that, like Holger, had studied at Chalmers to become civil engineers, I instead entered the Medical School in my hometown of Göteborg. Though I completed my five and a half years of basic medical courses, I had already in my second year during the course in physiology been attracted by the engineering/biophysical aspects of neurophysiology, and I became associated with the neurophysiology section as a medical student in the late 1960s. While the lab was mainly focused on cat spinal cord neuronal circuitry (see The experimental environment), there were also occasional studies of nerve cell biophysical properties. In a study of dorsal spinocerebellar tract (DSCT) cells (Eide et al. 1969) they had noted that the firing of these cells induced by intracellularly injected current as well as the post‐spike afterpotentials differed substantially from that of motoneurons, indicating distinct mechanisms in firing regulation for these two nerve cell categories. I was asked whether I would be interested in looking further into this question, which I was. So, first as a medical student, then as a Ph.D. student, and later holding various temporary positions, my research in the 1970s was mostly focused on this question, often together with a visiting scientist with time to spare from his intended project. In essence, I showed that the difference in firing behavior between motoneurons and DSCT cells was just based on differences in magnitude, kinetics and summation properties of the same underlying control mechanism, likely a calcium‐activated K current, these differences being important in changing a neuron from an integrating cell (motoneuron) to a relay cell (DSCT), see e.g., (Baldissera and Gustafsson 1974a, 1974b, 1974c; Gustafsson 1979; Gustafsson and Jankowska 1976; Gustafsson, Lindström, and Takata 1978; Gustafsson, Lindström, and Zangger 1978; Gustafsson and Lipski 1979; Gustafsson and Zangger 1978). At this time the department kept the authors in alphabetical order.

Following Holger's return to Göteborg my primary aim in collaborating with him was to extend these studies on firing regulation to an in vitro preparation using CA1/CA3 pyramidal cells. Thus, at the start of our in vitro slice experiments together I had had more than 10 years of experimental work on nerve cells, making my first intracellular recordings already in the late 1960s, but none related to synaptic transmission. During the period described here, 1981 to 1986, my in vivo work was still ongoing e.g., (Fetz and Gustafsson 1983; Gustafsson, Katz, and Malmsten 1982; Gustafsson and McCrea 1984; Gustafsson and Pinter 1984a, 1984b, 1984c; Gustafsson et al. 1986). In 1986 I was awarded Anders Jahre’s Nordic Prize in Medicine (University of Oslo, Norway) for younger redsearchers (below 40 years of age) for my “biophysical studies of nerve cell function”.

## 3 The Experimental Environment

As indicated above, neurophysiology in Göteborg in the 1970s consisted of in vivo studies mainly focused on the cat spinal cord. My thesis supervisor, Anders Lundberg had in the early 1960s, after post‐doctoral studies at Rockefeller Institute (N.Y.) under Lorento de Nó in 1949/1950, and as an invited guest researcher in Canberra with Eccles (1956/1957), created a research milieu in Göteborg that was not only renowned for its work on the spinal cord neuronal circuitry and its control but also for its high level of electronic/mechanical instrumentation. There was a steady stream of visiting scientists and Ph.D. students, some of which subsequently built illustrious careers, either staying on within the lab (like Elzbieta Jankowska) or later establishing their own labs elsewhere (like Sten Grillner).

To set up an in vitro laboratory in this environment came with both blessings and challenges. As a vibration isolation table Holger improvised from the existing equipment, using a table designed for cat experiments and placing it in boxes filled with sand, creating a double tabletop with well pumped‐up small dimension bicycle tubes interposed. He had acquired an “Oslo chamber” but since no pumps were available, we had to resort to gravity‐fed flow, which made drug application a bit tricky in the absence of recirculation. As can be noted from our publications, drugs were therefore often applied from a micropipette as droplets onto the surface of the slice. After some practice, this mode of application was easily managed without affecting recording/stimulation conditions. A definite upside of the in vivo environment was that much time and effort had been made in Göteborg to create custom‐built devices allowing mechanical stability for positioning both stimulation and recording electrodes, as well as for the equipment used to control stimulation parameters. In this digital era, I cannot but long for the ease of that manual analog control.

## 4 LLP Versus LTP

The paper published by Holger and me in early 1985 was in fact not on LTP induction but on LLP (long‐lasting potentiation) induction. LLP was the term used by Bliss and Lömo in their paper in 1973 (Bliss and Lomo 1973), the first proper description of this phenomenon, and it was also used in Oslo when Holger was there. And true to this Scandinavian tradition, LLP was used by Holger and me in most of our articles referred to here. It can, however, be noted that even in their 1973 paper Bliss and Lömo occasionally referred to it as long‐term potentiation, and LTP became also used by other contemporary authors (Alger and Teyler 1976; Douglas and Goddard 1975), LTP apparently also being an acronym more pleasing for an Anglo‐Saxon tongue. In fact, if we are to believe Tim Bliss the acronym LLP is a tongue twister for an Anglo‐Saxon (Bliss 2025), which is not the case for the Scandinavian tongue. Nevertheless, after the arrival at our lab of an Anglo‐Saxon tongue in the shape of Cliff Abraham in 1985 (after his post‐doc in Goddard's laboratory) we shifted from LLP to LTP during 1986, not wanting to be the odd men out. Other names, such as long‐term enhancement (McNaughton et al. 1978), have also been used. In this essay I will use LTP irrespective of the name used by the cited author(s).

## 5 Cooperativity

When Holger and I set out in the early 1980s it was understood that the increased synaptic response underlying LTP was specific to the activated synapses (Andersen et al. 1980, 1977). In contrast to the previously known short‐lasting synaptic plasticity processes, LTP induction appeared to depend on the number of activated synapses (Bliss and Gardner‐Medwin 1973). This cooperativity requirement for LTP induction became more systematically studied on both the perforant path synapses onto dentate granule cells (PP‐DG synapses) (Levy and Steward 1979; McNaughton et al. 1978) and the Schaffer collateral connections to CA1 pyramidal cells (CA3‐CA1 synapses) (Barrionuevo and Brown 1983; Lee 1983). While this co‐activity was often found associated with action potential activity in conformity with a Hebb synapse, a closer look revealed that spikes were found to be neither sufficient nor necessary for LTP induction. For example, activation of somatically projecting inhibitory pathways could nullify the action potential output produced by a strong excitatory action without affecting the LTP induction (Douglas et al. 1982). Adding an antidromic spike train to a tetanic afferent activation to increase the spike activity of the CA1 neurons did not affect the amount of LTP produced (Lee 1983). While not excluding the possibility of cooperative action through the release of some chemicals from the co‐active synapses, the common idea was rather a cooperative action on the postsynaptic side, i.e., the more co‐active synapses the larger the local depolarization in the dendritic shaft. This local depolarization might subsequently induce LTP by e.g., triggering calcium spikes invading the activated spines (Levy and Steward 1983). However, a paper by Holger together with McNaughton and Barnes challenged this notion (Wigström et al. 1982). They found that a large somatic hyperpolarization applied during an afferent tetanization by an injected current pulse was unable to affect the amount of LTP produced. The authors' interpretation of this result was that any integration of co‐activity was of a chemical rather than of an electrical nature.

## 6 The Inhibition‐Free Slice

I think Holger felt a bit taken aback by the above result and our joint work on the hippocampal slice started with work unrelated to LTP, such as electrical properties of CA1/CA3 pyramidal cells involved in firing regulation (Gustafsson, Galvan, et al. 1982; Gustafsson and Wigström 1981a, 1981b, 1983). For example, in collaboration with a group from Munich, we described the existence of a transient outward current in a mammalian neuron, the so‐called A current, strongly affecting the excitability of these cells (Gustafsson, Galvan, et al. 1982). However, I remember after returning from a brief stint in Munich in early 1982, Holger telling me that he had started to look at LTP after adding a GABA_A_ receptor antagonist to the bath solution and obtained interesting results. In his neural network models, Holger saw dendritic inhibition as an integral part in the control of synaptic plasticity. It was well established at this time that activation of the Schaffer collaterals in the CA1 region not only results in a monosynaptic EPSP but also in a dendritically located feed‐forward disynaptic IPSP (Alger and Nicoll 1982). So, despite the negative result described above, Holger had thought it well worth trying to at least see what happens when this inhibition is blocked.

In retrospect, it was obvious that studying LTP in the absence of inhibition is a win‐win situation. Inhibition can thereby be excluded from any observed changes in the test synaptic response, and an electrical versus chemical explanation for cooperativity was tested without any change in the number of co‐active excitatory synapses. I don't recall whether any tests were made for the anticipated appearance of seizure‐like activity after GABA_A_ receptor blockade. Anyhow, for this “inhibition‐free” slice, a cut between CA3 and CA1 was made, and the concentrations of calcium and magnesium in the bath solution were both raised to decrease membrane excitability, the increased magnesium also reducing the increase in release probability given by the increased calcium. The introduction of this “inhibition‐free” hippocampal slice preparation became very rewarding for us, and this preparation henceforth became a most useful tool in the study of LTP.

The outcome of this blockade for LTP induction was dramatic. A normally ineffective single 10‐impulse train (at 50 Hz) now produces a substantial LTP in the CA3‐CA1 synapses, even a 2‐impulse train resulting in some LTP. These results were published early in 1983 as a letter to Nature (Wigström and Gustafsson 1983a) and later as a full report (Wigström and Gustafsson 1985a). Even more dramatic results were found with respect to the PP‐DG synapses. It had been difficult to induce LTP at all in these synapses using the in vitro slice preparation, essentially all data up to this time coming from in vivo experiments. Our experiments revealed a strong feedforward inhibition that, when pharmacologically blocked, easily allows for substantial LTP induction (Wigström and Gustafsson 1983b). I seem to remember that Cliff Abraham came to visit us, and later worked with us, based on hearing about this result on the PP‐DG synapses. For us, the fact that the same afferent stimulation when allowed to produce a greater dendritic depolarization, so greatly facilitated LTP induction, appeared to strongly support an electrical rather than a chemical integration of synaptic co‐activity. So, why did a large somatic hyperpolarization fail to affect LTP induction in the article Holger authored with McNaughton and Barnes (Wigström et al. 1982)? I would think that had these authors reflected upon the much larger leakiness of the dendritic membrane during a tetanus‐induced synaptic barrage of EPSPs/IPSPs than during a single test EPSP, they would not have drawn any conclusion from these experiments. However, I fear that that article (Wigström et al. 1982) proposing a chemical rather than an electrical integration might have had a role in the fate of our Nature submission a few years later.

## 7 NMDA Receptor Involvement

There was an additional bonus using the “inhibition‐free” slice we had not anticipated. Because very brief trains can be used to induce LTP, our attention was drawn to the exact nature of the field response during such trains. We observed a slowly developing negativity of the field potential during the train, giving the impression of a dendritic regenerative event not observed in control solution (Wigström and Gustafsson 1983a, 1984). In fact, in these experiments, we had a loudspeaker turned on (as I was used to from in vivo experiments), and we imagined that whenever we heard the specific sound associated with the appearance of that negativity, an LTP followed. Thus, an additional dendritic depolarizing, perhaps even regenerative, process (synaptic or non‐synaptic) seemed to take place in the inhibition‐free slices as a possible correlate to LTP induction.

Now, assuming that cooperativity is explained by the level of dendritic depolarization, what accounts for the input specificity? At this time, it was thought that an increase in spine head calcium concentration might be a possible ultimate trigger for the LTP induction (Eccles 1983). But, if so, which is the physical connection between the level of dendritic depolarization, spine head calcium, and input specificity? Why should calcium accumulate only in active synapses, or, if accumulating in all synapses, only act in active synapses? At this time assorted data from other research groups had started to pile up. Using iontophoretic application, glutamate receptors of the NMDA type were found to be present on CA1 pyramidal cells, the opening of these calcium‐permeable NMDAR channels being voltage‐dependent (Dingledine 1983). The application of NMDAR antagonists had also been found to somehow impair LTP induction (Collingridge et al. 1983; Harris et al. 1984) and, alas, we found the slowly developing negativity to be blocked by an NMDAR antagonist (Wigström and Gustafsson 1984). After some time, we realized that if these NMDAR channels were co‐localized with the glutamate receptor channels responsible for the normally observed EPSP (the non‐NMDAR channels), a simple explanation for input specificity would exist since the calcium influx would automatically be restricted to the active synapses. I was initially somewhat refractory towards this idea as I could not see how self‐potentiation could be avoided. Why did not every single synaptic activation result in NMDAR activation, and hence in LTP, because of the large spine membrane depolarization provided by the non‐NMDA EPSP component? In our proposal paper that came out early 1985 (Wigström and Gustafsson 1985b), we simply postulate that the NMDAR channels would have to have slow kinetics and be unable to be opened until that spine depolarization was essentially over.

To our knowledge, no one had at that time described a synaptic potential mediated by NMDAR channels. Studies of CA1‐CA3 and PP‐DG synapses had suggested that only non‐NMDA receptors were involved in the synaptic response (Collingridge et al. 1983; Crunelli et al. 1982). Taking advantage of the fact that the voltage dependence of the NMDAR channels had been found to result from a voltage‐dependent block by magnesium ions (Mayer et al. 1984; Nowak et al. 1984), we made a “magnesium‐free” bath solution. We found the fEPSP in this solution to have an additional component with slow onset and long duration (Wigström et al. 1985). Using intracellular recording, we examined this EPSP at positive membrane potentials to remove its voltage dependence. A variation in the stimulation strength to cause a large range of EPSP peak amplitudes showed that the two EPSP components increased in parallel (Wigström and Gustafsson 1986). It would thus appear that these NMDARs are synaptic receptors that might be co‐localized with the non‐NMDA receptors, as we had proposed. I don't know what made this co‐localization concept stick so quickly. When Bekkers and Stevens described co‐localization in synapses to cultured hippocampal neurons examining “minis” (Bekkers and Stevens 1989), they introduced this co‐localization as being a central assumption for LTP and, based on their reference list, Holger and I were already pre‐history. In fact, it was seen almost as an anomaly when synapses with only one receptor type were found (so‐called AMPA‐silent synapse, or AMPA‐labile as we say in Göteborg (Groc et al. 2006)). Anyhow, the apparent rapid acceptance of this concept meant that we didn't feel obliged to provide more data on this issue than that published in connection with a meeting in Paris (Gif Lectures in Neurobiology; Synaptic regulation and plasticity) (Wigström and Gustafsson 1986).

There remained a potential worry, pointed out by Cliff Abraham, that the NMDAR activation during a brief tetanus simply serves as a cooperativity factor. Thus, the block of LTP by an NMDAR antagonist might just be a consequence of a reduced dendritic depolarization, like a smaller number of co‐active synapses, rendering the depolarization now below the threshold for LTP. This argument seems to carry weight when LTP is induced by an afferent tetanus where the depolarization is only achieved by synaptic activity. However, when the depolarization is primarily provided by injected current, and the synaptic involvement only consists of a single EPSP (as in the experiments described below), this argument seems to me to carry much less weight. To further counteract this argument, we used an almost “magnesium‐free” (0.1 mM) bath solution that would allow the NMDAR channels to open at subthreshold membrane potentials. This bath solution allowed for a substantial facilitation of LTP induction despite a virtual absence of postsynaptic firing during the brief train stimulation (Huang et al. 1987). In my mind, this result strongly supports that NMDAR activation, not dendritic depolarization per se, is the critical factor in LTP induction. However, I remember Cliff Abraham not being particularly impressed by these counterarguments, seeing them as being too indirect.

## 8 Pairing Experiments

Nonetheless, the stage was now set to test our proposed scenario for LTP induction by pairing single EPSPs with the postsynaptic depolarization timed to occur during the NMDAR component. Considering the failure to affect LTP induction by intracellular somatic current pulses (Wigström et al. 1982), Holger was a bit weary of using this technique. Instead, we started by repeatedly pairing single fEPSPs with brief (often 3‐impulse) 50 Hz trains to a separate set of afferents, both set of afferents in the stratum radiatum. The pairing procedure (Gustafsson and Wigström 1986) resulted in an LTP that was a sizeable fraction of that obtained by strong tetanization of that input, and that was fully occluded if preceded by such tetanization. Importantly, we found that LTP is also obtained with the conditioning train stimulation positioned in the stratum oriens, i.e., the conditioning depolarization must have passed the cell body to affect the test synapses in stratum radiatum. I recall Holger being quite relieved by this result, implying that current injected into the soma should be able to induce LTP. As described in the beginning of this article, LTP was indeed induced by postsynaptic activity produced purely electrically, if the injected current pulse was properly timed to the EPSP. A version of the article rejected by Nature was published in Acta Physiologica Scandinavica (Wigström, Gustafsson, Huang, and Abraham 1986) followed by a full paper in the Journal of Neuroscience (Gustafsson et al. 1987). In agreement with previous cooperativity studies, somatic action potentials were neither sufficient nor necessary. Thus, when paired with a single EPSP, the injected current had to produce more than 5 spikes during its duration of100 ms (i.e., spikes were not sufficient), and the injection of a local anesthetic blocking spike activity failed to block LTP (i.e., spikes were not necessary). In essence, the experimental validation of our proposed scenario for the associative induction of the Hebbian‐like hippocampal LTP was completed.

In general agreement with our results with current injection, Kelso et al. showed that pairing a weak afferent tetanus with postsynaptic activity generated by injected current resulted in LTP (Kelso et al. 1986). As in our case, postsynaptic spike activity was not necessary; injection of a local anesthetic blocking spike activity did not block LTP, indicating these synapses to be “pseudo‐Hebbian” rather than Hebbian. However, the manner dendritic depolarization was coupled with LTP induction was not explored. In a paper also published this year (Sastry et al. 1986), the authors showed, using a pairing protocol like ours (single EPSP and injected current), LTP to be induced in this associative manner. The first author, who had visited our lab in 1984 when we had started doing pairing experiments, did not interpret this result according to our scenario but rather seemed to favor a postsynaptic/presynaptic association process. In fact, in a previous study (Sastry et al. 1984), he had suggested that an NMDAR blocker does not block LTP but masks it. Such an action of these blockers was refuted by us (Wigström, Gustafsson, and Huang 1986).

With the advent of whole‐cell recordings in the early 1990s, a variant of our pairing‐protocol for LTP induction emerged in which the low frequency activation was paired with a steady membrane depolarization to close to zero membrane potential. Unwittingly, this procedure had also created a more optimal situation for evaluating the role of NMDAR activation in LTP induction. In this experimental situation, the observed blockade of LTP induction by an NMDAR antagonist (Isaac et al. 1995) is not likely related to a removal of NMDAR activation as a cooperativity factor. I would think that even Cliff Abraham would be satisfied by this argument.

## 9 Concluding Remarks

To wrap up this story, I will address some additional points concerning this Hebbian‐like LTP in consideration of the specific protocols for its induction that we have used.

As in vitro experiments, our studies have been conducted under non‐physiological conditions, but I do not think our “inhibition‐free” slices have introduced any further non‐physiology. Instead, I would find it surprising if not most synaptic learning in fact takes place under conditions of disinhibition, allowing it to be produced as rapidly and strongly as in our slices.

All potentiation induced in this Hebbian‐like manner is not LTP, i.e., lasting in a stable manner at least some 30–60 min, as is usually the criterion in slice preparations. Instead, at weaker activations it could vanish within a few minutes as a Hebbian‐like short‐term potentiation (Gustafsson et al. 1987).

Our induction protocol had also made it possible to evaluate the time required for LTP (for a population of synapses) to start to express itself, with minimal interference with other forms of plasticity (Gustafsson et al. 1989). LTP (at 30°C) had a latency of a few seconds and a growth phase of 15–20 s. Considering the doubling of these values when cooling to 25°C, they would accordingly at least be halved at body temperature. That is, in a behavioral test situation, synapses would be expected to have become potentiated within 10 s, or less.

A synapse population activated by a single pairing event displayed a rather modest LTP, about 10% (Gustafsson et al. 1989). However, given that the synaptic input to become potentiated is delivered in the form of a brief burst (Lisman 1997), LTP can work as a one‐shot potentiation, given sufficient postsynaptic activity. For example, applying just a single 5‐impulse tetanus to PP‐DG synapses, the test EPSP can increase to 50% above baseline, a very sizeable LTP (Hanse and Gustafsson 1992).

Concerning the pairing experiments, I think it is easy to overlook the fact that any strong depolarization that takes place when glutamate is still bound to the NMDA receptor will result in the opening of these channels and in calcium influx as well as currents that contribute to firing of the postsynaptic cell. In intracellular recordings, keeping the membrane potential at +30 mV, we found a sizeable NMDAR‐mediated EPSP component remaining at 200 ms after the onset of the single EPSP (Wigström and Gustafsson 1986). In the pairing experiments using depolarizing current pulses, there are also examples of LTP induction using pulses delivered 200 ms after the EPSP onset (Gustafsson et al. 1987). Thus, before implicating other mechanisms for the associative binding than that outlined here, the time window for the NMDAR‐mediated EPSP that follows the test EPSP/EPSPs should be known. It can be noted that a time window of about 200 ms was used with some success in a modeling study for spatial map formation (Blum and Abbott 1996).

Which is the actual role of somatic spike activation, the very essence of “Hebbianess”? After all, for the use of neurons as building blocks in “cell assemblies,” the temporal coincidence of input and output activity is of essence. And while in various experimental situations firing of the postsynaptic cell has been found neither necessary nor sufficient for LTP induction, under natural conditions, the generation of LTP may be highly correlated with postsynaptic firing even were there no physical coupling between the two (McNaughton et al. 1978). Considering the now revealed nature of the postsynaptic condition for LTP induction, some degree of physical coupling of the two may exist, especially if the somatic spike activity results in back‐propagation.

Nevertheless, it should be noted, somewhat ironically, that our quest for the associative device for Hebbian‐like LTP was performed in a region where somatic spike activity may be of limited consequence for synaptic learning. For example, in the CA1 region the association for learning is rather that between synaptic inputs arriving at the distal apical dendrite (SLM region) and proximal apical dendrite (SR region), respectively (Hasselmo 2005). That is, the device for LTP induction described here may have evolved for the temporal association between input events rather than between a synaptic event and somatic spike activity.

## Conflicts of Interest

The author declares no conflicts of interest.

## Data Availability

Data sharing not applicable to this article as no datasets were generated or analysed during the current study.
